# 

**Published:** 1886-10-30

**Authors:** 


### The Hospital.

An Institution, Family, and Parochial Journal of Hospitals, Asylums, and all Agencies for the Care of the Sick, Criticism and News.

SATURDAY, OCTOBER 30TH, 1886.

### NOTICE TO CORRESPONDENTS.

All correspondence must be written on one side of the paper only.

We cannot undertake to return MSS. not used.

**Communications**, whether intended for insertion or for private information, must be authenticated by the names and addresses of the writers, not necessarily for publication.

**Local papers** containing reports or news paragraphs **should be marked.**

It is especially requested that early intelligence of **local events of interest**, or which it may be thought desirable to bring under notice, may be sent direct to the Editor.

Communications respecting editorial matters should be addressed to the Editor, Norfolk House, Norfolk Street, Strand, London, W.C.

### "THE HOSPITAL" CHARITY BOX.

Mr. Frank Shapley, medical officer to the Sidcup Hospital, in entering as a competitor for the Prize Competition, expresses a hope that the institution with which he is connected may receive a small share of the funds obtained for the hospitals from this column. It is impossible to say at present to what extent, if at all, funds will result from the competition we have instituted. We desire it to be understood that prizes will be awarded irrespective of the number of competitors, and that an additional set of prizes will be distributed in any week in which the number of competitors exceeds 150. If, as we understand, a good income can be made by private individuals who run word-puzzles for their own advantage through the daily papers, we cannot but believe that, when The Hospital "Charity Box" gets known, the number entering will amount to hundreds instead of to tens, as at present. Whenever a competition results in a tie, as it recently did, the second and third prizes, or the first and second prizes as the case may be, will be added together and divided between those who tie. This, it has been pointed out, is only fair, seeing that, by merely dividing the prize for which there is a tie, you enable a competitor, who under other circumstances would not be placed, to gain a prize, which is undesirable and unfair.

---

OUR "Charity Box" is intended to afford a little practical help to the hospitals. Each week we propose a word for "anatomical dissection," and the first 150 competitors who extract the largest number of words from it receive the following prizes :—

| | |
|---|---|
| 1st .. .. .. | One Guinea. |
| 2nd .. .. .. | Half a Guinea. |
| 3rd .. .. .. | Five Shillings. |

The above Prizes will be repeated for every additional 150 competitors who may enter each week.

FOURTH COMPETITION.

Compose the largest number of words you can from "THE HOSPITAL."

*Observe:* Proper names, abbreviations, foreign and obsolete words, and repetitions are barred.

Write only on one side of the paper, and total your words.

Append your real name and address, adding whether Mr., Mrs., or Miss, etc.

All replies, addressed "Charity Box," THE HOSPITAL, Norfolk House, Norfolk Street, W.C., must arrive by Friday, November 5th. Results will appear Saturday, Nov. 13th.

*Every Competitor is required to cut out and enclose with his list the "Charity Box" Coupon* (see advertisement pages), *and a shilling entrance fee* (postal orders only).

The proceeds resulting from each "Charity Box" competition will be funded until the end of December 1886. The total sum available for distribution, and the names of the hospitals selected for donations, will appear on Saturday, January 8, 1887. The distribution of the proceeds will be at the Editor's sole discretion.

NOTE.—The words "the" and "hospital," do *not* count in this competition ; plurals admissible.

RESULT OF THIRD COMPETITION

The "John Hunter" Competition has resulted as follows :—

| | Score. |
|---|---|
| *Mr. H. Tetley*, Laleham, near Staines .. | 101—1st Prize. |
| *Mrs. A. S. Sheldon*, Parkside House, Macclesfield .. .. .. .. .. .. | 98—2nd Prize. |
| *Mr. A. E. Cooper*, 10, South Bailey, Durham .. .. .. .. .. | 97—3rd Prize. |

We must again ask competitors not to extend their lists by the inclusion of barred words, as their excision gives unnecessary trouble. This has reduced totals in several cases.

We hardly thought it necessary to point out that no letter must be used twice or more in a word, unless it occurs twice or more in the word to be "dissected." One competitor last week had her score of 134 words reduced by no less than 50, almost entirely through the repetition of letters. In the present competition the only letters which may occur twice in a word are "t" and "h."

---

### BUSINESS COMMUNICATIONS AND ADVERTISEMENTS.

All communications concerning business matters, non-delivery of THE HOSPITAL, etc., should be addressed to the Manager, at the Publishing Offices, 30 and 32, Sardinia Street, Lincoln's Inn Fields, London, W.C.

**Cheap Advertisements** of the "Wanted" class, including those for situations, of Twenty-four Words or under, are charged *1s.* an insertion, or three insertions for *2s. 6d.*, but must be paid for in advance. Each additional line of Eight Words is *4d.*

**Employers requiring assistants** are charged for one insertion of Twenty-four Words or under, *2s. 6d.*, or *6s.* for three insertions. Each additional line of Eight Words is *1s.*

**Advertisements** for insertion in the weekly issue of THE HOSPITAL **must be delivered** at the Publishing Offices **not later than 11 a.m. on Wednesdays.**

Cheques and Post Office Orders to be made payable to Messrs. WHITING & Co. Subscribers and Advertisers are requested **not to send stamps.**

### THE CHILDREN'S CORNER.

"Little Mary" says she has obtained for me five friends who now all read the "Children's Corner." "Freddie" writes me a nice little letter, the first, he tells me, that he has ever addressed to an editor. But, despite this fact, "Freddie" is evidently an observant little boy, for he remarks that he "always thought" that newspaper editors said "we," and not "I," as I do. Yes, "Freddie," you are quite right. Newspaper men and kings do, as a rule, use the mighty "we," though your "Puzzle Editor" adopts a less assuming pronoun.

### OUR PASTIMES.

**Children's Quarterly Prize Competition.**

| | |
|---|---|
| Began .. .. .. .. | 2nd Oct., 1886 |
| Ends .. .. .. .. | 25th Dec., 1886 |

PRIZES.

| | |
|---|---|
| 1st .. .. .. .. | Thirty Shillings |
| 2nd .. .. .. .. | Twenty " |
| 3rd .. .. .. .. | Ten " |
| 4th .. .. .. .. | Five " |

will be awarded to the four competitors, who shall have sent in, during the thirteen weeks, the largest number of correct answers to our pastimes.

**Fifth Competition.**

No 21. Tell me, if you can, the difference between a governess and a lady who just manages to lose her train.

No. 22. Compose a sentence of not more than nine words, which shall contain *all* the letters of the alphabet.

No. 23. Translate yy u r yy u b
i c u r yy 4 me.

No. 24. Take away three sides of the following figure, so as to leave just three perfect squares, and no odd sides over.

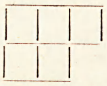

No. 25. What word is there of eight letters, yet contains only one vowel ?

No. 26. A blind beggar had a brother. The brother was injured by an accident and died. Now, who can tell me what relation the blind beggar was to the brother before the brother died ?

**Results of Third Competition.**

No. 10 I wonder how it is that only "Alsie" and "Charlie Powell" have guessed this ! "Bed" is the answer.

No. 11. "Retsibsi" has alone guessed the Latin puzzle, which reads :—"Will(i)am runs against the mantelpiece."

The Latin words require, as my little friends will see, rather a literal translation.

No 12. The "lights" of this are "Ship," "Phial," "Pets," and "Health"; of course no one doubts that what everybody wants to read must be THE HOSPITAL.

No. 13. This sentence reads : "Sir, between ourselves, you underrate my understanding" It was easily found out, I fancy.

No. 14 No one has guessed this. The monosyllable is *vague ;* omit the "v," and we get the dissyllabic word, "ague."

No. 15. The snail has puzzled most of my little readers. In 23 days, the snail will have climbed 23 half-feet, or, in other words, he will be within six inches of his goal. Now, when he enters on his 24th day's exploit, he will accomplish the remaining six inches in six hours, *and when he reaches the top, he will not, of course, fall down again.* Hence the correct answer is, not 24 days, but 23 days, 6 hours.

All right—None.
Five right—None.
Four right—Diosota, Alsie, Freddie.
Three right—Retsibsi, Ubique, and Katie King.
Two right—George Collins, C. Tynget, Little Mary.
One right—Charlie Powell, Daisy, Bismarck, and Nonsuch.

Answers, having the actual name and address (to which a pseudonym may be added for publication), must have "The Children's" Coupon attached (see advertisement pages), addressed to the "PUZZLE EDITOR, THE HOSPITAL, Norfolk House, Norfolk Street, Strand, W.C.," not later than Thursday, 4th November. Results will appear in our issue of the 13th November.

### THE IN-PATIENTS' HOSPITAL DIARY.

*(Continued from p. 68.)*

The following Table gives the terms of admission, the names of the Physicians and Surgeons, and the days and hours of their attendance upon the In-patients at all the General Hospitals in the Metropolis, and at the Special Hospitals, in alphabetical order of Diseases, up to and including half the Ophthalmic Hospitals.

### EYE DISEASES.—*Continued.*

| Hospitals and Terms of Admission. | Medical Officers, and Days and Hours of Attendance. Physicians. | Surgeons. |
|---|---|---|
| Royal London Ophthalmic Hospital, Blomfield Street, Moorfields, E.C.<br>Admission free to the poor | Daily, 8 to 10 | Mr. J. Hulke<br>„ G. Lawson<br>„ J. Couper<br>„ W. Tay<br>„ J. Tweedy<br>„ Nettleship<br>„ A. Silcock<br>„ Gunn<br>„ Lang |
| Royal South London Ophthalmic Hospital, 6, St. George's Circus, Southwark, S.E.<br>Free to the necessitous poor; or by payment | Daily, 2 | Mr. Watson<br>„ McHardy<br>„ A. S. Morton<br>„ Mackinlay |
| Royal Westminster Ophthalmic Hospital, King William Street, W.C.<br>Free to accidents and the indigent poor; or by payment | Daily, 2 | Mr. Power<br>„ Frost<br>„ Rouse<br>„ Hartridge<br>„ Macnamara<br>„ Cowell<br>„ Juler |
| St. Bartholomew's Hospital, Smithfield, E.C. See p. 16 | Tu., Th., 1.30; M., Th., S., 2 | Mr. Power<br>„ Vernon |
| St. George's Hospital.<br>By Governors' letters. See p. 16 | M.. Th., 2; F., 1.15; W., S., 2 | Mr. B. Carter<br>„ Frost |
| St. Mary's Hospital.<br>By Governors' letters. See p. 16 | Tu., F., 9.30 | Mr. A. Critchett<br>„ H. Juler |
| St. Thomas's Hospital, Westminster Bdg. Rd., S.E. See p. 16 | M., Th., 2 | Mr. Nettleship |
| University College Hospital.<br>By Governors' letters. See p. 16 | M., Tu., Th., F., 2 | Mr. Tweedy |
| West London Hospital.<br>By Governors' letters. See p. 16 | Tu., Th., S., 2 | Mr. B. Vernon<br>„ H. Dunn |
| Westminster Hospital.<br>By Governors' letters. See p. 16 | M., Th., 2.30 | Mr. Cowell |
| Western Ophthalmic Hospital, 153, MaryleboneRd., W. By small payments or In-patients' letter | Dr. Miller<br>„ Collins<br>„ Wheatly | Mr. C. Jones<br>„ Buxton<br>„ Barrett |

### HEART, PARALYSIS, EPILEPSY, AND DISEASES OF THE NERVOUS SYSTEM GENERALLY.

| | | |
|---|---|---|
| Hospital for Epilepsy and Paralysis, etc., 32, Portland Ter., Regent's Park Rd., N.W.<br>By payment, but free to the necessitous poor | Dr. J. Althaus<br>„ A. Bennett<br>„ G. Ogilvie<br>„ G. Laycock | Mr. J. Bloxam<br>„ A. P. Gould |
| National Hospital for the Paralysed and Epileptic, Queen Square, W.C.<br>By subscriber's letter | Dr. Ramskill<br>„ Radcliffe<br>„ H. Jackson<br>„ Buzzard<br>„ Ormerod<br>„ Beevor<br>M., Tu., W., F., 2.30 | Mr. W. Adams<br>„ Horsley |
| West End Hospital for Diseases of the Nervous System, 73, Welbeck Street, W.<br>By subscriber's letter | Dr. Tibbitts<br>„ Stretch-Dowse<br>„ S. Armitage | Mr. S. Benton |

### ORTHOPÆDIC CASES AND DEFORMITIES OF BODY GENERALLY.

| | | |
|---|---|---|
| City Orthopædic Hospital, 27, Hatton Garden, E.C. Free | Tu., F., 2 | Mr. E. J. Chance<br>„ J. Addison |
| National Orthopædic Hospital, 234, Great Portland Street, W. Free | Dr. C. Beevor | Mr. F. R. Fisher<br>„ W. Roeckel |
| Royal Orthopædic Hospital, 297, Oxford Street, W.<br>By Governor's letter | Daily, 1 | Mr. Broadhurst<br>„ H. Reeves<br>„ C. Read<br>„ W. Balkwill<br>„ H. F. Baker |
| St. Bartholomew's Hospital, Smithfield, E.C. Free. See p. 16 | M., 2.30 | Mr. Walsham |

### SKIN, DISEASES OF.

| Hospitals and Terms of Admission. | Medical Officers, and Days and Hours of Attendance. Physicians. | Surgeons. |
|---|---|---|
| British Hospital for Diseases of the Skin (West Branch), 61, Great Marlborough St., W.<br>By payment; necessitous free | M., 2; W., 7; F., 2 | Mr. B. Squire<br>„ G. Gaskoin |
| Charing Cross Hospital.<br>By Governor's letters. See p. 16 | Dr. Sangster | M., 2 |
| Guy's Hospital.<br>By payment of 3*d*. each | Dr. Pye-Smith<br>„ Hale-White | Tu., 12 |
| Hospital for Diseases of the Skin, 52, Stamford Street, S.E.<br>Free and by payment | Dr. W. Cottle<br>„ E. J. Payne | Mr. Waren Tay |
| King's College Hospital.<br>By Governor's letter. See p. 16 | Dr. Duffin | F., 1 |
| London Hospital.<br>By Governor's letter. See p. 16 | M., Th., 1.30 | Mr. Couper |
| Middlesex Hospital.<br>By Governor's letter. See p. 16 | Dr. R. Liveing | F., 4 |
| National Institution for Diseases of the Skin, 227, Gray's Inn Road, W.C.<br>By payment, or free on certificate of a medical man or minister | Dr. B. Meadows | M., Th., 6 |
| North-West London Hospital.<br>By Governor's letter. See p. 16 | Dr. J. Stowers | Tu., 1.30 |
| Paddington Green Children's Hospital. Free. See p. 32 | Dr. T. C. Fox | S., 9.15 |
| St. Bartholomew's Hospital. Free. See p. 16 | F., 1.30 | Mr. F. Cripps |
| St. George's Hospital.<br>By Governor's letter. See p. 16 | Dr. Cavafy | W., 2 |
| St. Mary's Hospital.<br>By Governor's letter. See p. 16 | M., Th., 9.30 | Mr. Morris |
| St. Thomas's Hospital.<br>By letter. See p. 16 | Dr. Payne | W., 1.30 |
| University College Hospital.<br>By Governor's letter. See p. 16 | Dr. Crocker | W., 1.30; S., 9.15 |
| Westminster Hospital.<br>By Governor's letter. See p. 16 | Dr. T. C. Fox | W., 1.30 |

### STONE AND OTHER DISEASES OF URINARY ORGANS.

| | | |
|---|---|---|
| St. Peter's Hospital for Stone, Henrietta Street, W.C.<br>Admission free | .... | Mr. E. Fenwick<br>„ F. Edwards<br>„ Heycock |

### DISEASES AND IRREGULARITIES OF THE TEETH.

| | | |
|---|---|---|
| Charing Cross Hospital.<br>By Governor's letter. See p. 16 | M., W., F., 9.0 | Mr. Fairbank |
| Dental Hospital of London, Leicester Sq., W.C.<br>Free to the poor, together with any operative assistance that may be immediately necessary. For special operation by Governor's letter | Daily, 9 to 11 | Mr. Canton<br>„ Gregson<br>„ Hepburn<br>„ S. Bennett<br>„ Underwood<br>„ Woodhouse<br>„ Hern<br>„ Matheson<br>„ Parkinson<br>„ Read<br>„ Rogers<br>„ Truman |
| German Hospital.<br>Free to Germans. See p. 16 | Th., 10 to 11 | Mr. A. P. Rebou<br>„ C. West |
| Great Northern Central Hospital. Free. See p. 16 | W., 1.30 | Mr. E. Keen |
| Guy's Hospital.<br>On payment of 3*d*. See p. 16 | Tu., Th., F., 12.30 | Mr. Moon<br>„ Pedley |
| Hospital for Consumption, Brompton.<br>By Governor's letter | W. and when wanted | Mr. C. J. Noble |
| Hospital for Sick Children, Gt. Ormond Street, W.C. | W., 9 | Mr. Cartwright |

### DISEASES AND IRREGULARITIES OF THE TEETH.—*Continued.*

| Hospitals and Terms of Admission. | Medical Officers, and Days and Hours of Attendance. Physicians. | Surgeons. |
|---|---|---|
| King's College Hospital. By Governor's letter. See p. 16 | Tu., F., 10 | Mr. Cartwright |
| London Hospital. By Governor's letter. See p. 16 | Tu., 9 | Mr. Barrett |
| Metropolitan Free Hospital. Free. See p. 16 | Tu., Th., S., 9 | Mr. G. Curnock |
| Middlesex Hospital. By Governor's letter. See p. 16 | M., F., 9.30; W., 9 | Mr. Bennett, „ Hern |
| National Dental Hospital, 149, Gt. Portland Street, W. By Governor's letter | .... | Mr. Williams, „ H. Rose, „ A. F. Canton, „ F. H. Weiss, „ T. Gaddes, „ A. Smith, „ W. G. Weiss, „ W. Humby, „ G. Bradshaw |
| National Hospital for Disease of Heart. By Governor's letter | W., 11 | Mr. J. Fitzgerald |
| National Orthopædic Hospital, Gt. Portland Street, W. | .... | Mr. H. Harris |
| New Hospital for Women, Marylebone Road | .... | Mr. H. Apperley |
| North-West London Hospital. By Governor's letter | F., 9 | Mr. W. A. Maggs |
| Paddington Green Children's Hospital. Free. See p. 32 | W., 9 | Mr. C. Cottrell |
| Royal Free Hospital, Gray's Inn Road, W.C. | .... | Mr. H. Harris |
| Royal Hospital for Children and Women. By Governor's letter | F., 1.30 | Mr. Whitehouse |
| St. Bartholomew's Hospital. Free. See p. 16 | Tu., F., 9 | Mr. Ewbank, „ Paterson |
| St. George's Hospital. By Governor's letter. See p. 16 | Tu., S., 9 | Mr. Winterbottom |
| St. Mary's Hospital. By Governor's letter. See p. 16 | Dr. H. Hayward | W., S., 9.30 |
| St. Thomas's Hospital. Free. See p. 16 | Tu., F., 10 | Mr. Truman |
| University College Hospital. By Governor's letter. See p. 16 | W., 9.30 | Mr. Hutchinson |
| Victoria Hospital for Children. See p. 32 | S., 9 a.m. | Mr. F. Fox |
| West London Hospital. By Governor's letter. See p. 16 | .... | Mr. H. L. Albert |
| Western Ophthalmic Hospital. See p. 87 | .... | Mr. W. Forsyth |
| Westminster Hospital. By Governor's letter. See p. 16 | Dr. Walker, W., 9.15 | Mr. Smale, S., 9.15 |

### THROAT DISEASES.

| Hospitals and Terms of Admission. | Physicians. | Surgeons. |
|---|---|---|
| Central London Throat and Ear Hospital. See p. 32 | Mr. L. Browne, „ F. G. Hamilton, „ A. W. Orwin, „ J. D. Grant, „ P. S. Jakins | M., W., Th., S., 2; Tu., F., 5 |
| Evelina Hospital for Sick Children, Southwark Bridge Road, S.E. | .... | Mr. R. D. Pedley |
| Great Northern Central Hospital. Free. See p. 16 | W., 2 | Mr. Watson |
| Guy's Hospital. On payment of 3*d*. | Th., 1 | Mr. Symonds |
| Hospital for Diseases of the Throat. By payment, but free to the necessitous poor | Daily, 1.30 | Mr. Wokes, „ T. Hovell, „ G. Stoker, „ Fenton-Jones, Daily, 2 |
| Infirmary for Consumption, 26, Margaret Street, W. See p. 16 | .... | |
| King's College Hospital. By Governor's letter. See p. 16 | Tu., Fri., 10 | Mr. Cartwright |
| Metropolitan Ear and Throat Infirmary. See p. 32 | Daily, 2.30 | .... |
| Middlesex Hospital. By Governor's letter. See p. 16 | Tu., 9 | Mr. Hensman |
| Municipal Throat and Ear Infirmary. See p. 32 | Dr. G. Holmes, „ J. A. Hatch | M., W., F., 10 to 12 |
| North-West London Hospital. By Governor's letter. See p. 16 | M., 3 | .... |
| St. Bartholomew's Hospital. Free. See p. 16 | F., 2.30 | Mr. Butlin |

### THROAT DISEASES.—*continued.*

| Hospitals and Terms of Admission. | Physicians. | Surgeons. |
|---|---|---|
| St. George's Hospital. By Governor's letter. See p. 16 | Th., 2 | Mr. Whipham |
| St. Mary's Hospital. By Governor's letter. See p. 16 | M., Th., 9.30 | Mr. A. T. Norton |
| St. Thomas's Hospital. Free. See p. 16 | Dr. Semon | Tu., F., 1.30 |
| University College Hospital. By Governor's letter. See p. 16 | Dr. Poore | Th., 1.30 |
| Westminster Hospital. By Governor's letter. See p. 16 | W., 9 | .... |

### DISEASES OF WOMEN (OBSTETRIC CASES, ETC.).

| Hospitals and Terms of Admission. | Physicians. | Surgeons. |
|---|---|---|
| Charing Cross Hospital. By Governor's letter. See p. 16 | Dr. J. W. Black, „ A. Routh | Tu., F., 1.30 & 2 |
| Chelsea Hospital for Women, Queen's Elm, Fulham Road. For reception of gentlewomen in reduced circumstances, and respectable poor women. Payment from 10*s*. 6*d*. a week. Gratuitous treatment to poor on recommendation of a Governor | Dr. J. H. Aveling, „ A. W. Edis, „ F. Barnes | M., Tu., W., F., 2 |
| East London Hospital for Children and Dispensary for Women. See p. 32 | Dr. E. Smith, „ H. B. Donkin, „ H. R. Crocker, „ J. A. Coutts, „ D. Williams | Mr. A. Cæsar, „ R. W. Parker, „ L. A. Dunn |
| German Hospital, Dalston Lane, E. | Dr. Rasch | M., Th., 9 |
| Great Northern Central Hospital. Free. See p. 16 | Dr. F. Barnes | W., 2 |
| Grosvenor Hospital for Women and Children. See p. 32 | M., Tu., Th., S., 2 to 3 | .... |
| Guy's Hospital. Free without letter (Uterine Wards). See p. 16 | Dr. Galabin, „ Horrocks | M., Tu., F., 1.30 |
| Hospital of St. John and St. Elizabeth, 50, Great Ormond Street, W.C. For females suffering from advanced or long-standing disease | Dr. Constable, „ Tegart | .... |
| Hospital for Women, Soho Sq., W. | Dr. Carter, Tu., F., 9.30; M., W., Th., F., S., 2 | Mr. H. A. Reeves, M., Th., 4 |
| King's College Hospital. By Governor's letter. See p. 16 | Dr. Playfair | Tu., Th., S., 2 |
| London Hospital, 281, Whitechapel Road, E. | Dr. Herman, „ Lewers | M., Tu., Th., F., 2 |
| Metropolitan Free Hospital. Free. See p. 16 | Dr. A. Venn | W., 2 |
| Middlesex Hospital. By Governor's letter. See p. 16 | Dr. Edis | Tu., F., 1.30 |
| New Hospital for Women, 222, Marylebone Road. By subscriber's letter. All the physicians are women | Mrs. Anderson, „ Atkins, „ Marshall, „ de la Cherois, Daily, 1 to 3; S., 9 to 10 | Mr. Lawson, „ Norton, „ Smith, „ Meredith, Daily, 1 to 3; S., 9 to 10 |
| North-West London Hospital. By Governor's letter, or payment | W., 1.30 | .... |
| Royal Free Hospital, Gray's Inn Road. Free. See p. 16 | Dr. T. C. Hayes | S., 2. |
| Royal Hospital for Children and Women. By Governor's letter, and payment of 1*d*. per week | Dr. Duncan, „ Haddon, „ Park, „ Dakin, Daily | Mr. Jacobson, „ Day, M., Th. |
| St. Bartholomew's Hospital. Free. See p. 16 | Dr. M. Duncan, „ Godson | Tu., Th., S., 2; W., S., 9 |
| St. George's Hospital. By Governor's letter. See p. 16 | Dr. Champneys | Tu., F., 2 |
| St. Mary's Hospital. By Governor's letter. See p. 16 | Dr. Meadows, „ H. Jones | M., Th., 9.30; M., Th., 1.30 |
| St. Thomas's Hospital. Free. See p. 16 | Dr. Gervois, „ Cory | Tu., F., 2; W., S., 1.30 |
| Samaritan Free Hospital for Women and Children. See p. 32 | Daily, 12 to 2 | .... |
| University College Hospital. By Governor's letter. See p. 16 | Dr. G. Hewitt, „ J. Williams | Tu., F., 2 |
| West London Hospital. By Governor's letter. See p. 16 | Dr. A. Venn, „ Moullin | M., S., 2 |
| Westminster Hospital. By Governor's letter. See p. 16 | Dr. Potter, „ Grigg | Tu., F., 2, 9 |

☞ Secretaries will oblige by communicating necessary corrections from time to time.

